# Liver-related events and mortality among elderly patients with advanced chronic hepatitis C treated with direct-acting antivirals

**DOI:** 10.1371/journal.pone.0217052

**Published:** 2019-06-03

**Authors:** Iria Rodríguez-Osorio, Alvaro Mena, Héctor Meijide, Luis Morano, Manuel Delgado, Purificación Cid, Luis Margusino, José Domingo Pedreira, Ángeles Castro

**Affiliations:** 1 Grupo de Virología Clínica, Instituto de Investigación Biomédica de A Coruña (INIBIC)-Complexo Hospitalario Universitario de A Coruña (CHUAC), Sergas, Universidade da Coruña (UDC), A Coruña, Spain; 2 Unidad de Enfermedades Infecciosas, Servicio de Medicina Interna, Complexo Hospitalario Universitario de A Coruña (CHUAC), Sergas, A Coruña, Spain; 3 Servicio de Medicina Interna, Hospital Quironsalud, A Coruña, Spain; 4 Unidad de Patología Infecciosa, Servicio de Medicina Interna, Hospital Álvaro Cunqueiro, Complexo Hospitalario Universitario de Vigo, Vigo, Spain; 5 Servicio de Aparato Digestivo, Complexo Hospitalario Universitario de A Coruña (CHUAC), Sergas, A Coruña, Spain; 6 Servicio de Farmacia, Complexo Hospitalario Universitario de A Coruña (CHUAC), Sergas, A Coruña, Spain; Universita degli Studi di Pisa, ITALY

## Abstract

**Background:**

Direct-acting antivirals (DAAs) are effective in patients aged ≥65 years. However, little is known about the effects of DAAs on survival, liver decompensation and development of hepatocellular carcinoma (HCC).

**Objective:**

To compare the incidence of liver-related events and mortality between patients aged ≥65 and <65 years.

**Methods:**

Prospective study comparing patients aged ≥65 and <65 years treated with DAAs. The incidence of liver-related events and mortality, and HCC was compared between age groups.

**Results:**

Five hundred patients (120 aged ≥65 and 380 aged <65 years) were included. The incidence of liver-related events was 2.62 per 100 patient-years (py) in older and 1.41/100 py in younger patients. All-cause mortality was 3.89 and 1.27/100 py in older and younger patients, respectively. The respective liver-related mortality rates were 1.12 and 0.31/100 py. In patients with cirrhosis (stage F4), all-cause mortality (*P* = 0.283) and liver-related mortality (*P* = 0.254) did not differ between groups. All five liver-related deaths were related to multifocal HCC. The incidence of HCC was 1.91 and 1.43 per 100 py in the older and younger groups, respectively (*P* = 0.747). The diagnosis of HCC was 8 months after the end of treatment.

**Conclusions:**

The incidence of liver-related events and liver-related mortality was low in older people treated with DAAs and was similar to that in younger patients. The extra mortality in people aged ≥65 years treated with DAAs seems to be secondary to non-liver-related causes. These results support the utilization of DAAs in patients aged ≥65 years.

## Introduction

Since the appearance of direct-acting antivirals (DAAs), patient subgroups previously not included in HCV treatment now have the option of receiving treatment. DAAs against HCV have been shown to be effective and tolerable in elderly patients (aged ≥65 years) in clinical trials and in real-life cohorts [1–5]. The rate of a sustained virological response at 12 weeks (SVR12) after the end of treatment (EOT) is >90%.

This high SVR12 has led to unquestionable benefits such as the reduced mortality and liver decompensation rates, but most studies are from the IFN years, when all special populations were underrepresented, especially those aged ≥65 years. There is a lack of more data analysing the favourable impact of SVR12 with the use of IFN-free DAAs.

The aim of this study was to evaluate the incidence of liver-related events and survival rates in patients aged ≥65 years treated with DAAs. We compared the rates of liver-related events, mortality and liver-related mortality between patients aged ≥65 years and <65 years with similar liver damage treated similarly.

## Materials and methods

### Ethics statement

The study protocol was reviewed and approved by the Medical Ethics Committee of Galicia (register code 2015/164) and was conducted in accordance with the Declaration of Helsinki and the STROBE Statement [6]. A database was created, and all data were anonymized before analysis.

### Study protocol

This was an observational prospective cohort study conducted between August 2012 and October 2017, in two tertiary Hospitals of the Northwest of Spain: University Hospital of A Coruña (CHUAC) and the University Hospital Álvaro Cunqueiro Vigo (CHUVI). Those two hospitals have an influence population in 2016, approximately, of 600000 citizens each; of them, 25% were older than 65 years old. The study protocol included two phases, first phase of the study, previously reported [7], was conducted between August 2012 and October 2015. The aim of the first phase of the study was to response questions about efficacy and tolerability of DAAs in elderly patients (≥65 years old). The inclusion criteria of this first phase was being in follow up in those two Hospital, elderly patient (≥65 years old), treated with DAAs regimens and signed the informed consent. Second phase of the study, was conducted between November 2015 and October 2017. The primary end point of this second phase was the incidence of liver-related events occurring after EOT in elderly patients previously included in the first phase of the study and to compare them with a sample of younger patients (<65 years old) hepatitis C chronic infected patients in follow up in those two Hospitals. Liver-related events were defined as at least one of the following: liver decompensation, HCC diagnosis, liver transplantation or liver-related mortality. The same patient could have more than one liver-related event; for such patients, time was censored at the first episode. The secondary clinical endpoints were HCC incidence and all-cause mortality. Data were compared between patients aged ≥65 years and <65 years. The inclusion criteria of this second phase for elderly patient was: being included in the first phase of the study and to signed the informed consent; while for younger patients was: being over 18 years old and younger than 65 years old, being in follow up in liver clinic of these two Hospitals, being treated with DAAs regimens during the study period and signed the informed consent. All patients were in clinical follow up of liver clinics, where laboratory tests were performed for every visit and they were included in a HCC screening program with ultrasound every 6 months.

### Statistical analyses

Continuous variables are reported as mean ± standard deviation (SD) or median (IQR), as indicated. For dichotomous and categorical variables, absolute numbers and percentages were computed. Student’s *t* test and chi-squared analysis were used for analyses, after checking the normality of the distribution.

The times to different events were calculated from EOT to the event, and the follow-up (FU) was censored on 31 October 2017. Liver-related events, mortality rates and incident HCC rates were calculated per 100 patient-years (100 py) with 95% CIs. Kaplan–Meier curves were compared using log-rank tests between the two age groups. Statistical analysis was performed using IBM SPSS Statistics (version 21.0).

## Results

Five hundred patients were included: 120 patients aged ≥65 years (30.8% older than 75 years) and 380 nonelderly patients, all without HIV infection. The median time of FU was 24 months (IQR, 16–26). Table 1 shows the main characteristics, liver-related events and mortality rates.

**Table 1:** Characteristics of the study population.

	Elderly (≥ 65 years) *n* = 120	Younger (<65 years) *n* = 380	Elderly (>75 years) *n* = 37
**Demographic characteristics**			
**Age, years (mean ± SD**^**a**^**)**	72.6 ± 7.4	51.2 ± 7.1	78.8 ± 3.3
**Gender, n (%)**			
**Men**	57 (47.5)	298 (78.4)	20 (54.1)
**Virological characteristics and liver status**			
**Genotype, n (%)**			
**1a**	7 (5.8)	102 (26.8)	2 (5.4)
**1b**	100 (83.3)	133 (35.1)	33 (89.2)
**1 Unknown subtype**	8 (6.8)	0	1 (2.7)
**2**	3 (2.5)	15 (3.9)	1 (2.7)
**3**	1 (0.8)	70 (18.4)	0
**4**	1 (0.8)	60 (15.8)	0
**Stiffness**^**b**^**, kPa, (median, IQR)**	16.0 (10.0–21.4)	12.5 (9.9–20.0)	16.9 (12.0–21.9)
**F2, n (%)**	18 (15.0)	72 (18.9)	5 (13.5)
**F3, n (%)**	25 (20.8)	146 (38.4)	4 (10.8)
**F4, n (%)**	77 (64.2)	162 (42.7)	28 (75.7)
**SVR12**^**c**^**, n (%)**	111 (99.1)	326 (97.4)	30 (100)
**Clinical and liver-related events, n (%)**			
**Liver-related events**	7 (5.8)	9 (2.4)	4 (10.8)
**Any tumour**	9 (7.5)	13 (3.4)	4 (10.8)
**HCC**^**d**^	5 (4.2)	9 (2.4)	2 (5.4)
**Liver decompensation**	4 (3.3)	4 (1.1)	1 (2.7)
**Hydropic decompensation**	2 (1.6)	2 (0.5)	0.0
**Upper bleeding**	1 (0.8)	1 (0.3)	0.0
**Encephalopathy**	1 (0.8)	1 (0.3)	1 (2.7)
**All-cause mortality**	10 (8.3)	8 (2.1)	6 (16.2)
**Liver-related death**	3 (2.5)	2 (0.5)	1 (2.7)
**Liver transplantation**	0.0	4 (1.1)	0.0

^a^SD: standard deviation

^b^measured using the Fibroscan

^c^SVR12: sustained virological response after 12 weeks from end of treatment

^d^HCC: hepatocellular carcinoma.

**Fig 1:**
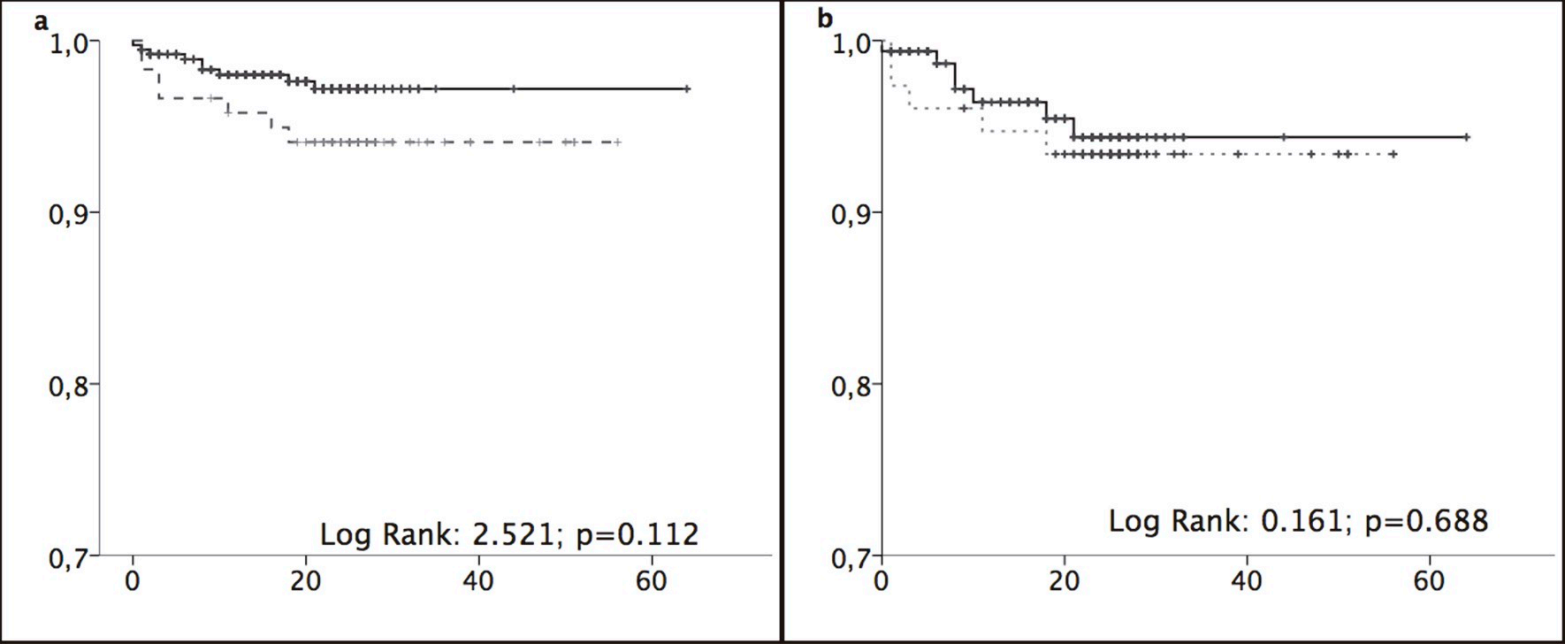
Liver-related events. (a) Liver-related events. (b) Liver-related event in stage F4 patients. Kaplan–Meier curves were compared using log-rank tests between patients aged ≥65 years (black lines) and <65 years (grey dotted lines). Abscissa: time in months. Ordinate: cumulative survival.

Liver-related events occurred in 5.8% and 2.4% of patients aged ≥65 years and <65 years, respectively, after a median of 3.5 months and 8.2 months FU treatment, respectively. The incidence rates were 2.62/100 py (1.28–5.31) and 1.41/100 py (0.74–2.65) in the older and younger groups, respectively (Fig 1 and 1A). Analysis of only patients with cirrhosis (stage F4) showed higher incidence rates in the older and younger groups: 3.45/100 py (1.59–7.31) vs. 2.63/100 py (1.28–5.36) (Fig 1 and 1B).

**Fig 2:**
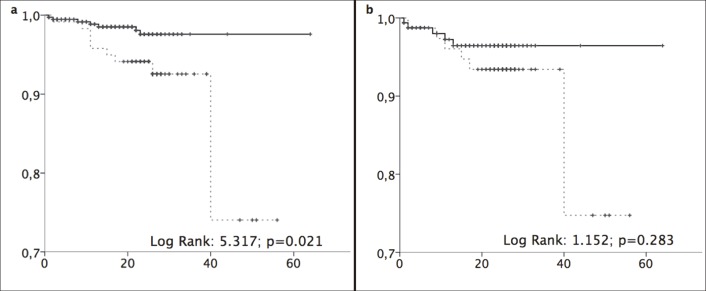
All-cause mortality. (a) All-cause mortality. (b) All-cause mortality in stage F4 patients. Kaplan–Meier curves were compared using log-rank tests between patients aged ≥65 years (black lines) and <65 years (grey dotted lines). Abscissa: time in months. Ordinate: cumulative survival.

All-cause mortality rates were 3.89/100 py (2.09–7.25) and 1.27/100 py (0.64–2.51) in the older and younger groups, respectively (Fig 2 and 2A). Patients died after a median FU treatment of 11 months in both groups. Mortality was more than three times higher in the older group (6.96/100 py) than in the younger group (2.21/100 py).

**Fig 3:**
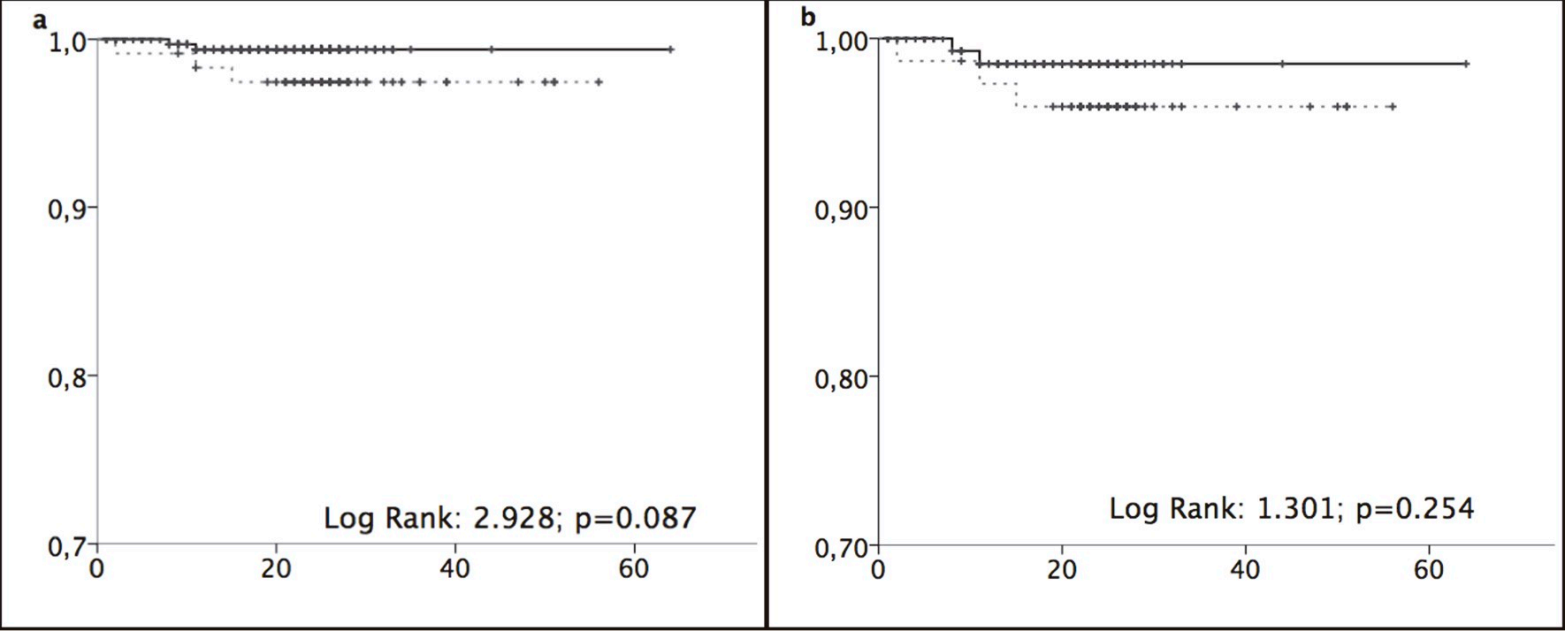
Liver-related mortality. (a) Liver-related mortality. (b) Liver-related mortality in stage F4 patients. Kaplan–Meier curves were compared using log-rank tests between patients aged ≥65 years (black lines) and <65 years (grey dotted lines). Abscissa: time in months. Ordinate: cumulative survival.

Liver-related mortality rates were 1.12 (0.38–3.25) per 100 py and 0.31 (0.09–1.14) in the older and younger groups, respectively (Fig 3 and 3A). Comparison of F4 patients only showed no significant differences between age groups in all-cause mortality (Fig 2 and 2B) or liver-related mortality (Fig 3 and 3B). All five liver-related deaths were related to multifocal HCC.

All patients were included in the HCC screening programme, and the tumour diagnosis was made during the FU according to radiological and clinical criteria. The percentage of patients with HCC staged using the Barcelona Clinic Liver Cancer staging in the older group was early stage (A) 40.0%, intermediate stage (B) 20.0% and advanced stage (C) 40.0%. HCC was present in 2.4% of patients in the younger group; the staging was stage A, 66.7%; stage B, 11.1%; and stage D, 22.2%.

The incidence of HCC was 1.57/100 py (0.93–2.65); incidence was 1.91/100 py and 1.43/100 py in the older and younger groups, respectively. In F4 patients, the incidence was 2.87/100 py (1.23–6.55) and 3.38/100 py (1.79–6.20) in older and younger patients, respectively. The median time of HCC diagnosis was 8 months (3–15) after EOT and did not differ between groups.

Other cancers besides HCC were colon adenocarcinoma, pancreatic adenocarcinoma, oropharynx tumour and cholangiocarcinoma in older patients, and colon adenocarcinoma, lung adenocarcinoma, non-Hodgkin lymphoma and brain tumour in younger patients.

## Discussion

The arrival of DAAs has enabled eradication of HCV in subgroups of patients with poor access to IFN-based therapies. During the IFN years, older patients were undertreated because of toxicity and poor efficacy. The benefits of the high SVR in younger patients are reduced mortality and liver-related events (including HCC development). Inclusion of patients eligible for IFN treatment in previous studies may have introduced a selection bias that magnified the beneficial effects of IFN plus ribavirin on the SVR; however, recent data of DAAs corroborate the benefits of an SVR [8,9].

In our study, the incidence of liver-related events was 1.6 episodes/100 py in both older and younger patients and did not differ significantly between age groups. This is similar to that reported by Van der Meer (1.1/100 py), who included only young IFN-treated patients treated, about 50% of whom had cirrhosis [10] In our study, the incidence in F4 patients was >3/100 py and did not differ between age groups.

All-cause mortality is better than liver-related mortality as a clinical end point when analysing the effects of an SVR because the latter can include indirect causes related to cirrhosis. It has been reported before, as in the Veterans Affairs Clinical Registry published by McCombs, that the risk of death was reduced by 45% in patients whom achieved SVR [11]. Our data on liver-related mortality in older patients are consistent with the higher all-cause mortality rate in older populations. As expected, all-cause mortality was higher in older than in younger patients in our study, but this extra risk related mainly to the inclusion of patients aged >75 years and secondarily to non-liver-related events. We did not analyse the effects of an SVR in this population for two reasons: the high efficacy of treatment (>90%) and our aim to evaluate the treatment strategy in older people to answer the question of whether to treat.

Most of the liver-related mortality was related to HCC. Age is known as an independent indicator of progression of liver fibrosis [12]. Guo et al reported a poorer prognosis for HCC patients aged ≥65 years than in younger patients, but older patients were diagnosed later and were treated less aggressively [13]. And in our data all liver-related mortality in elderly was related to HCC.

The reported incidence of HCC in patients treated with DAAs varies. Some studies show little effect on or an increase in the incidence, especially for early recurrences [14, 15] However, larger studies have shown a reduced HCC incidence after DAA treatment of similar magnitude to that obtained with IFN-based therapies (>70% reduction in HCC risk) [16]. In our study, the global HCC incidence was 1.57/100 py, which is close to the 1.9/100 py reported recently by Backus et al in a large cohort of US veterans with advanced liver disease and 35.3% of patients aged ≥65 years [17].

These data are relevant in the current Spanish scenario with universal access to treatment for all patients. This has been shown to be cost-effective in Europe, where the cost of DAA treatment is €15,000, and the incremental cost-effectiveness ratio is €9,107.60/QALY [18]. The median age in the study of Kondili was 59 years, although about 25% of patients were aged ≥65 years and 12% were aged >75 years. Cost-effectiveness data are lacking in patients aged ≥65 years [19] With the current cost of DAAs in Spain, the incremental cost-effectiveness ratio has been reduced to around €2,000/QALY. This seems to justify the treatment of people with a reasonable life expectancy regardless of age or degree of fibrosis.

This study has limitations. The sample size was small, there was a short FU and we were unable to identify factors related to prognosis. It was possible that HCC was present but undiagnosed when the patients started DAAs, although all cirrhotics were in an HCC screening programme. As noted above, we have not analysed the effects of an SVR. Finally, the higher proportion of F4 in the elderly group may lead to higher rate of events in them, although the specific analysis in F4 patients can minimize the bias.

## Conclusions

In conclusion, the incidence of liver-related events and liver-related mortality were similar in patients aged ≥65 years and <65 years. This supports the universal use of DAAs in this population.

## Supporting information

S1 Data Set Anonymized data set. (SAV)
